# Transition to electronic medical records improves efficiency and reach of antimicrobial stewardship service in an Australian tertiary hospital setting

**DOI:** 10.1017/ash.2025.58

**Published:** 2025-03-24

**Authors:** George P Drewett, Danni Miatke, Mani Suleiman, Taksh Singh Mann, Bruce Lin, Saliya Hewagama

**Affiliations:** 1 Department of Infectious Diseases, Northern Health, Epping, VIC, Australia; 2 Faculty of Medicine, Dentistry and Health Sciences, University of Melbourne, Parkville, Victoria, Australia; 3 Pharmacy Department, Northern Health, Epping, VIC, Australia; 4 Research Development and Governance Unit, Northern Health, Epping, VIC, Australia

## Abstract

We examined the effect of transition to electronic medical records on the antimicrobial stewardship service (AMS) in our healthcare service, finding significant increases (*P* < 0.001) in the number and type of prescribed restricted antimicrobials identified for review, number of patients seen, and AMS intervention significance post transition.

## Introduction

Antimicrobial stewardship services have become an indispensable tool in the provision of safe and appropriate antibiotic prescription and in combating the spread of antimicrobial resistance in hospital settings. In Australia, the transition from paper-based to an electronic medical record (EMR) in the public hospital sector has been underway for several years, with the majority of tertiary and quaternary healthcare settings now operating an EMR.

EMR is potentially transformative to the practice of antimicrobial stewardship service (AMS) in the hospital setting. The effects of EMR on AMS have been studied in the Australian setting at other centers where EMR has been implemented^1–3^, finding better identification of patients on antibiotics with better efficiency, increased review of antibiotics prescribed, and reduced time spent on AMS ward round. Further, reduced use of restricted antibiotics, and better documentation of antibiotic indication have also been shown. EMR has even been used to automate stewardship prompts to encourage appropriate prescribing with marked success.^4^

Our article examined the effect of the transition from paper-based to electronic medical records in an Australian tertiary healthcare setting, prospectively evaluating the effect of this transition on the workload, function, and effectiveness of the hospital AMS program.

## Methods

We conducted a quasi-experimental pre-post study using routine data collected as part of AMS rounds and activities recorded on *Guidance MS*, an Australian web-based antimicrobial decision support program, designed and supported by the National Centre for Antimicrobial Stewardship.^5^ Data regarding patient age, admitting specialty, restricted antimicrobial prescribed, therapy appropriateness, AMS advice, and intervention significance were collected. Definitions for therapy appropriateness and intervention significance, and a full list of restricted antimicrobials at our institution, can be found in the *Supplementary materials*. In addition, the following AMS ward round (WR) data were collected during each ward round by the AMS pharmacist attending the round: total patients flagged for AMS review; number of patients actually reviewed on WR; time spent on AMS activities pre-WR; time spent on WR: time spent post-WR; and time spent in the intensive care unit (ICU). Flags for AMS review were generated via a prescriber-initiated approval code on *Guidance MS* at time of prescription or by ward pharmacists during dispensing, and reviewed by the AMS pharmacist pre-WR.

The data were collected over two six-week periods prior to and following transition to EMR; 12th July to 28th August 2023 and 7th February to 26th March 2024. WR occurred thrice weekly, attended by an infectious diseases consultant and AMS pharmacist, during both study periods. Non-ICU patients were reviewed on the wards pre-EMR, and office-based post-EMR, with ICU patients reviewed in the ICU for both study periods. Transition from paper-based records to EMR occurred from the 5th to 13th September 2023. The five months from September 2023 to January 2024 were censored from our evaluation period to allow for any learning curve, “teething problems” or staff diversions associated with the transition.

We performed descriptive and statistical analysis using *Stata MP Version 18.0* (StataCorp, College Station, TX, USA). Chi-square or Fisher’s exact tests were used to test for statistically significant differences in categorical variables. For continuous variables, normality was tested with the Shapiro-Wilk test. Normally distributed variables are reported using mean and standard deviation, with differences being tested for statistical significance using t-tests. Non-normally distributed variables are reported using median and interquartile range, with the Mann-Whitney U test used to detect statistically significant differences.

Local governance approval was granted by Northern Health Office of Research (2023_non-HREC_06).

## Results

Compared with the pre-EMR study period, in the 6-week post-EMR implementation period there was a 48% increase in the total volume of antimicrobial prescriptions flagged for AMS review (458 pre-EMR vs 680 post-EMR). While these alerts increased across all admitting specialties, this difference was most pronounced in general surgery (64 vs 164 alerts, representing 14% vs 24% of all flags) and pediatrics (2 (0.4%) vs 17 (2.5%)). Accordingly, the overall distribution of admitting specialty units changed significantly (p < 0.001). The number and proportion of alerts from patients admitted to the intensive care unit (ICU) also increased significantly (6 (1.3%) vs 70 (10.3%), *P* < 0.001). A summary of the pre- and post-EMR AMS Guidance data is provided in Table 1. A summary of the pre- and post-EMR ward round data is provided in *Supplementary Table 1*.

**Table 1.** Guidance data pre- and post-EMR implementation

	Pre-EMR	Post-EMR	p-value
N	458	680	
Age, mean (SD)	**69.8 (18.6) (n = 458)**	**62.7 (20.9) (n = 680)**	**<0.001**
**Admitting specialty**			**<0.001**
General Medicine	199 (43.4%)	252 (37.1%)	
General Surgery	64 (14.0%)	164 (24.1%)	
Pediatrics	2 (0.4%)	17 (2.5%)	
Specialty Medicine	131 (28.6%)	183 (26.9%)	
Specialty Surgery	51 (11.1%)	56 (8.2%)	
Other	11 (2.4%)	8 (1.2%)	
**Ward: Intensive Care Unit**	**6 (1.3%)**	**70 (10.3%)**	**<0.001**
**Antimicrobial prescribed**			**<0.001**
Aciclovir (intravenous route)	9 (2.0%)	19 (2.8%)	
Azithromycin (intravenous route)	46 (10.0%)	15 (2.2%)	
Ceftriaxone	195 (42.6%)	368 (54.1%)	
Ciprofloxacin	13 (2.8%)	33 (4.9%)	
Meropenem	25 (5.5%)	25 (3.7%)	
Moxifloxacin	13 (2.8%)	10 (1.5%)	
Piperacillin-tazobactam	107 (23.4%)	126 (18.5%)	
Vancomycin	26 (5.7%)	30 (4.4%)	
Other	22 (4.8%)	49 (7.2%)	
Not Documented	2 (0.4%)	5 (0.7%)	
**Therapy Appropriateness**			
Optimal	76 (42.9%)	201 (46.4%)	0.47
Adequate	22 (12.4%)	63 (14.5%)	0.52
Suboptimal	64 (36.2%)	133 (30.7%)	0.19
Inadequate	8 (4.5%)	24 (5.5%)	0.69
Not Assessable	7 (4.0%)	12 (2.8%)	0.45
**Antimicrobial Already Ceased**	**263 (57.4%)**	**192 (28.2%)**	**<0.001**
Indication Documented	458 (100%)	674 (99.1%)	0.087
AMS Advice			
**Cease Antimicrobial**	**15 (3.3%)**	**45 (6.6%)**	**0.015**
Change to PO (Oral) route	60 (13.1%)	96 (14.1%)	0.66
**Change Dose**	**2 (0.4%)**	**26 (3.8%)**	**<0.001**
Change Spectrum	31 (6.8%)	39 (5.7%)	0.53
Broader	2 (0.4%)	6 (0.9%)	
Narrower	25 (5.5%)	30 (4.4%)	
Other	4 (0.9%)	3 (0.4%)	
**Change Duration**	**40 (8.7%)**	**34 (5.0%)**	**0.014**
Extend Approval	12 (2.6%)	35 (5.1%)	0.047
Plan for future management	10 (2.2%)	11 (1.6%)	0.51
Additional investigation suggested	29 (6.3%)	45 (6.6%)	0.90
Suggest referral to infectious diseases	25 (5.5%)	47 (6.9%)	0.38
**Intervention Significance**			**<0.001**
None	294 (64.2%)	321 (47.2%)	
Minor	28 (6.1%)	73 (10.7%)	
Moderate	132 (28.8%)	280 (41.2%)	
Major	4 (0.9%)	6 (0.9%)	

Caption: Pre-EMR (Electronic Medical Record): refers to data collected during pre-intervention time period; Post-EMR: refers to data collected during post-intervention time period; SD: Standard Deviation. Definitions for Therapy Appropriateness and Intervention Significance can be found in the Supplementary materials.

Alerts for most of the commonly prescribed restricted antimicrobials increased, although this was most pronounced for ceftriaxone (195 (42.6%) vs 368 (54.1%)) and ciprofloxacin (13 (2.8%) vs 33 (4.9%)). Alerts for meropenem, moxifloxacin, piperacillin-tazobactam, and vancomycin were similar pre- and post-EMR. Alerts for intravenous azithromycin decreased (46 (10%) vs 15 (2.2%)). There was a significant decrease in alerts for antimicrobials already ceased by the time of the AMS round review (57.4% vs 28.2%, *P* < 0.001). Of antibiotics prescribed ongoingly at the time of AMS review, the therapy appropriateness did not significantly differ.

Recommendations of the AMS service differed pre- and post-EMR, with increases in recommendations to cease the antimicrobial (3.3% vs 6.6%, *P* = 0.015) and change the dose (0.4% vs 3.8%, *P* < 0.001), and decreases in recommendations to change the duration (8.7 vs 5.0%, *P* = 0.014). Recommendations to change the route or spectrum of antimicrobials, or referral to infectious diseases, did not significantly differ. Overall, the significance of the AMS intervention differed pre- and post-EMR (*P* < 0.001) with a proportional decrease in reviews leading to no significant intervention (64.2% vs 47.2%).

**Figure 1.**
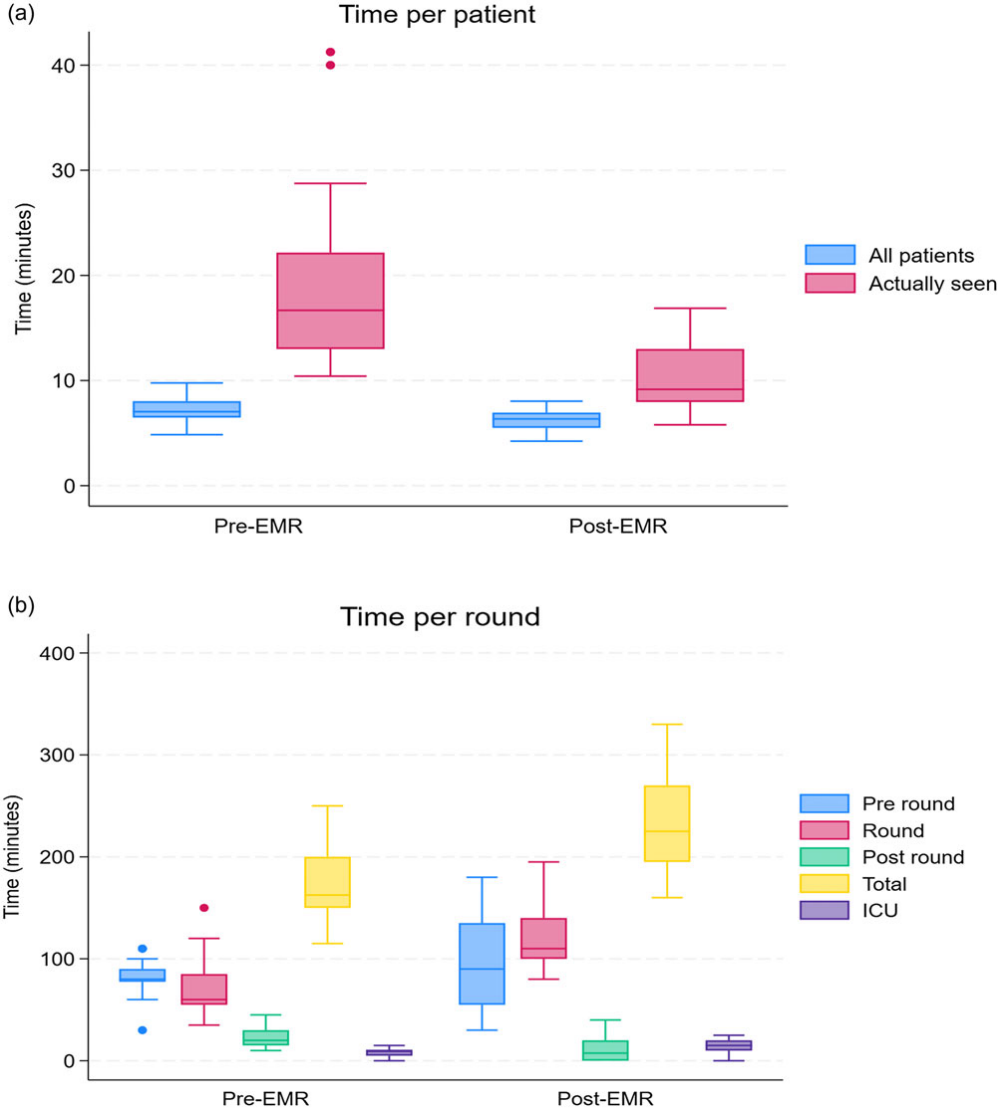
Time spent per patient (a) and ward round (b). Caption: Pre-EMR (Electronic Medical Record): refers to data collected during pre-intervention time period; Post-EMR: refers to data collected during post-intervention time period; All patients: Total patients flagged for AMS review; Actually seen: Patients reviewed on AMS WR; Pre-round: AMS pharmacist reviews flags and prepares ward round list; Round: AMS pharmacist and Infectious Diseases Consultant conduct WR; Post-round: AMS pharmacist finalizes WR recommendations and completes data entry; ICU: Component of AMS round conducted in the ICU/for ICU patients. Numerical time data can be found in *Supplementary Table 1*.

Post-EMR, there was a 51% increase in patients flagged for AMS review per WR (24.3 vs 36.8, *P* < 0.001), and a 124% increase in patients actually seen on WR (10.7 vs 24.0, *P* < 0.001). Total time spent on WR increased (175 vs 229 min, *P* < 0.001), although time spent per patient actually seen on WR significantly decreased (16.7 vs 9.2 min, *P* < 0.001), as shown in Figure 1.

## Discussion

Integrated technology to assist in AMS activity has been in use for over 2 decades^6^. Our experience demonstrates a significant effect of transition to EMR on AMS services in our hospital setting. Our findings suggest we were better able to identify patients prescribed restricted antimicrobials leading to dramatically increased review, in line with published data.^1,3^ This is likely due to the increased capacity to provide prospective audit and feedback on antimicrobial prescription.^7^

Previous studies have demonstrated an increase in antibiotic prescribing appropriateness following EMR intervention^8^, improvements in the efficiency of interventions and outcome reporting^9^, and easier identification of patients prescribed restricted antimicrobials^10^, which is reflected in our study. Paper medication chart-based prescribing is reliant on prescribers and ward pharmacists accurately seeking and documenting AMS approval, which is often user and unit dependent, as the significant increase in general surgical and pediatric AMS alerts post-EMR suggests. An EMR-based prescribing system with instantaneous access to prescriptions provides far better oversight of antimicrobial prescription and dispensing and a fairer representation of hospital usage.

Further, time spent on manual retrieval of paper-based medication charts and patient histories forms a significant part of a paper-based AMS round, reflected in the significant decrease in time spent per patient post transition to EMR. In addition, time spent walking between hospital wards was also eliminated post-EMR, further improving round efficiency.

The younger age of the post-intervention cohort is likely due to increased capture of antimicrobial use on the pediatric and surgical units, which have relatively younger populations. The pre- and post-EMR time periods occurred in different seasons; the pre-EMR period occurred during winter, while the post-EMR period occurred during summer and early autumn. Seasonality is known to influence antimicrobial prescribing, with increased antimicrobial use during winter months^11^. While this is a relevant confounder, this would be expected to reduce the effect of the EMR intervention in our study, and strengthens our findings of increased identification of antimicrobial prescription post-EMR, during a period where less antimicrobial use may be expected.

Unlike others’ experience where transition to EMR resulted in less time spent during WR^2^, as a result of the substantial increase in alerts generated post-EMR we found a significantly increased time spent performing AMS WR. This has implications for other AMS programs planning on transitioning to EMR in settings where prior paper medical charting and reliance on individually generated antibiotic approvals may incompletely capture actual prescribing behavior. This consideration would have been useful in planning AMS service resources and provision when considering the transition to EMR at our own institution.

In the long term, to achieve meaningful improvements in appropriate antibiotic prescribing on a hospital level, the increase in workload created by an EMR environment with more complete information capture will require more resourcing to adequately provide stewardship services.

## Conclusion

The transition from a paper-based to an EMR-based system had a dramatic effect on AMS in our healthcare setting, resulting in a more complete and useful AMS service. Despite significant efficiency gains, due to the dramatic increase in workload generated by a more accurate system for the identification and control of restricted antimicrobial prescription, time spent during AMS rounds significantly increased post-transition. It is imperative that adequate resources are available to ensure the ongoing provision of a quality AMS service in the high information capture environment provided by EMR.

## Supporting information

Drewett et al. supplementary material Drewett et al. supplementary material

## Supplementary material

For supplementary material accompanying this paper visit http://doi.org/10.1017/ash.2025.58. click here to view supplementary material

## Research transparency and reproducibility

All de-identified data is available by request to the corresponding author.
