# Supplementary material for: Transition to electronic medical records improves efficiency and reach of antimicrobial stewardship service in an Australian tertiary hospital setting
Source: Antimicrob Steward Healthc Epidemiol. 2025 Mar 24;5(1):e85. doi: 10.1017/ash.2025.58 (PMC11951236; doi:10.1017/ash.2025.58)
Supplement: Drewett et al. supplementary material [file S2732494X25000580sup001.docx]

# Supplementary material - Transition to electronic medical records improves efficiency and reach of antimicrobial stewardship service in an Australian tertiary hospital setting

Drewett et al 2025

Contents

[Supplementary table 1: Ward Round data pre- and post-EMR implementation 2](#_Toc190258482)

[National Antimicrobial Prescribing Survey Appropriateness Definitions 3](#_Toc190258483)

[Definition of AMS Intervention Significance 4](#_Toc190258484)

[List of Restricted Antimicrobials, Northern Health 5](#_Toc190258485)

## Supplementary table 1: Ward Round data pre- and post-EMR implementation

|  | Pre-EMR | Post-EMR | p-value |
| --- | --- | --- | --- |
| Ward rounds performed (N) | 20 | 18 |  |
| Patient data |  |  |  |
| Total Patients Flagged, mean (SD) | **24.3 (5.1) (n=20)** | **36.8 (6.9) (n=18)** | **<0.001** |
| Patients Post Pre-Round, mean (SD) | **10.7 (5.0) (n=20)** | **29.7 (6.9) (n=18)** | **<0.001** |
| Number Actually Seen, mean (SD) | **10.7 (5.0) (n=20)** | **24.0 (6.8) (n=18)** | **<0.001** |
| Time data (minutes) |  |  |  |
| Pre-round time, median (IQR) | 80.0 (77.5, 90.0) | 90.0 (55.0, 135.0) | 0.55 |
| Ward Round time, mean (SD) | **70.8 (30.7)** | **120.6 (32.0)** | **<0.001** |
| Post-round time, mean (SD) | 22.2 (10.3) | 10.8 (12.9) | 0.004 |
| Time spent in ICU, mean (SD) | 8.8 (4.6) | 13.1 (8.2) | 0.051 |
| Total time, mean (SD) | **175.0 (39.7)** | **229.1 (48.7)** | **<0.001** |
| Time per patient flagged, mean (SD) | **7.3 (1.3)** | **6.3 (1.0)** | **0.011** |
| Time per patient seen, median (IQR) | **16.7 (13.0, 22.2)** | **9.2 (8.0, 13.0)** | **<0.001** |

Caption: SD: Standard Deviation; IQR: Interquartile Range; Pre-EMR (Electronic Medical Record): refers to data collected during pre-intervention time period; Post-EMR: refers to data collected during post-intervention time period; Total patients flagged: Total patients flagged for AMS review; Patients Post Pre-Round: Patients identified for review following AMS pharmacist review of flagged patients; Number Actually Seen: Patients reviewed on AMS WR by Infectious Diseases consultant and AMS pharmacist; Pre-round: AMS pharmacist reviews flags and prepares ward round list; Ward Round: AMS pharmacist and Infectious Diseases Consultant conduct WR; Post-round: AMS pharmacist finalises WR recommendations and completes data entry; Time spent in ICU: Component of AMS round conducted in the ICU/for ICU patients.

## National Antimicrobial Prescribing Survey Appropriateness Definitions


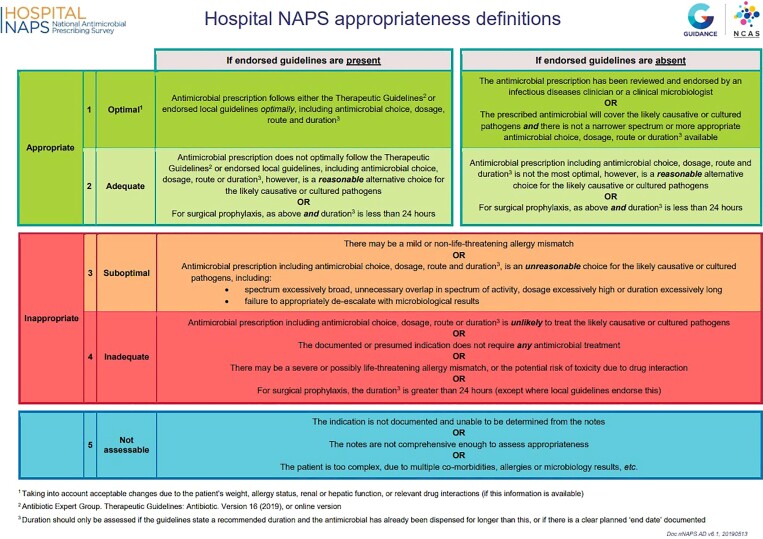


Courtesy: National Centre for Antimicrobial Stewardship. https://www.ncas-australia.org/

## Definition of AMS Intervention Significance

- **Mild**: No significant changes to current therapy required, but additional considerations or therapy optimisations available
  - Example: Recommendations around duration, potential additional investigations, minor therapy amendments
- **Moderate:**Change to therapy recommended to address Stewardship concerns, Guideline compliance and/or improve patient safety.
  - Example:  IV to PO step down, removal of PIVC, dosing amendments, change in spectrum of activity, targeting of therapy based on micro results
- **Major:**Urgent change to therapy required due to concerns with patient safety. Major interventions usually occur in instances when serious harm was likely to occur if the AMS team had not reviewed them.
  - Example: Missed diagnosis, severe allergy mismatch, severe micro-mismatch/ missed results, drug toxicity

## List of Restricted Antimicrobials, Northern Health

Aciclovir (IV)

Amikacin

Amphotericin (IV)

Anidulafungin

Azithromycin (IV)

Aztreonam

Baricitinib

Caspofungin

Cefepime

Cefotaxime

Ceftaroline

Ceftazidime

Ceftazidime-avibactam

Ceftriaxone

Ciprofloxacin

Colistin

Dapsone

Daptomycin

Ertapenem

Ethambutol

Fluconazole

Fosfomycin

Fusidic Acid

Ganciclovir

Gentamicin

Imipenem

Isoniazid

Linezolid

Meropenem

Molnupiravir (Inpatient use)

Moxifloxacin

Norfloxacin

Paxlovid (Inpatient use)

Pentamidine

Piperacillin-tazobactam

Posaconazole

Pristinamycin

Pyrazinamide

Quinupristin-dalfopristin

Remdesivir

Rifabutin

Rifampicin

Rifaximin

Teicoplanin

Tigecycline

Tobramycin (IV)

Valganciclovir

Vancomycin

Voriconazole
